# Rapid Construction of PVA@CDs/SiO_2_ Fluorescent/Structural Color Dual-Mode Anti-Counterfeiting Labels via Spray-Coating Method

**DOI:** 10.3390/polym16152211

**Published:** 2024-08-02

**Authors:** Wenjuan Liu, Ling Yang, Ting Li, Yuejun Liu, Zengming Tang, Shuyang Hu, Xionggang Wang, Haihu Tan

**Affiliations:** 1 College of Packaging and Materials Engineering, Hunan University of Technology, Zhuzhou 412007, China; m22080500012@stu.hut.edu.cn (W.L.); yangling2020@hut.edu.cn (L.Y.); m230805z1001@stu.hut.edu.cn (T.L.); yjliu_2005@126.com (Y.L.); 21404100112@stu.hut.edu.cn (S.H.); wangxionggang@hut.edu.cn (X.W.); 2 College of Life Science and Chemistry, Hunan University of Technology, Zhuzhou 412007, China; 14315@hut.edu.cn

**Keywords:** anti-counterfeiting label, carbon quantum dots, photonic crystal, silicon dioxide

## Abstract

A method of sequential spraying of polyvinyl alcohol with carbon quantum dots (PVA@CDs) aqueous suspension and SiO_2_ aqueous suspension is proposed to rapidly prepare multicolor dual-mode anti-counterfeiting labels. With the optimization of the concentration (15%) of colloidal microspheres in the SiO_2_ aqueous suspension as well as the spraying process parameters (spray distance of 10 cm, spray duration of 3 s, and assembly temperature of 20 °C), different-sized SiO_2_ microspheres (168 nm, 228 nm, and 263 nm) were utilized to rapidly assemble red, green, and blue photonic crystals. Furthermore, the tunable fluorescence emission of carbon quantum dots endows the labels with yellow, green, and blue fluorescence. The constructed dual-mode labeling was used to develop an anti-counterfeiting code with dual-channel information storage capabilities and also to create dual-mode multicolor anti-counterfeiting labels on various packaging substrates. This work provides a novel solution for anti-counterfeiting packaging and information storage.

## 1. Introduction

Novel anti-counterfeiting and information storage technologies supply effective solutions to avoid forgery and information leakage, which severely violate consumer rights and create risks for economic health. Dual-mode optical anti-counterfeiting labels record information using two different modes, featuring characteristics of complexity in replication and encryption, thus providing dual functions of anti-counterfeiting and information security. Combined with the fluorescent and structural color displays, dual-mode optical labels demonstrate concealed fluorescence and the challenging-to-replicate characteristics of the photonic crystal (PC) structure, indicating excellent application prospects [1,2].

The current methods for fabricating fluorescent PC labels can be generally divided into two routes. One route involves preparing fluorescent colloidal microspheres followed by their self-assembly to realize the fabrication of fluorescent/structural color dual-mode films. Fang et al. [3] synthesized a novel high-polymer PC microsphere P(HFBMA-co-GMA) containing hydrophobic functional groups and active epoxy groups. Then, the functional microspheres were utilized to prepare Janus-colored cotton fabrics with different colors and different hydrophilic properties on both sides after being spray-coated on polydopamine (PDA)modified cotton fabric. Chen et al. [4] employed dendrimer-shaped macromolecules as bridging grafts to incorporate fluorescent Cu-In-S/ZnS quantum dots (QDs) and SiO_2_ microspheres for constructing colloidal photonic crystals, which exhibit stable structural colors and fluorescence. Kuo and colleagues [5] assembled polystyrene (PS) microspheres into closely packed opaline photonic crystals using the dip-coating method. This periodic array of PS microspheres was employed as a rapid qualitative sensor for methanol and ethanol. Solvent samples placed on the PS photonic color-changing sensor were detected through color changes. Although this construction method can achieve stable performance of dual-mode optical labels, the preparation of fluorescent colloidal microspheres is still troublesome and not easy to implement. The other route involves introducing fluorescent materials (quantum dots [6,7], dyes [8,9], upconversion nanoparticles [10], and organometallic complexes [11]) into existing photonic crystal structures [12,13,14] to endow them with fluorescence characteristics. The fluorescence of fluorophores can be dramatically enhanced when the photonic bandgap (PBG) and fluorescent wavelengths overlap because of the Bragg reflection effect. For example, Chen et al. [15] dynamically adjusted the photoluminescence (PL) characteristics of a fluorophore by matching the shift of the photonic bandgap by tuning the reflection and PL wavelengths based on the bilayer structure during stretching. Alternatively, fluorescent materials can be embedded into photonic crystal structures (inverse opal structure; opal structure). Li et al. [16] permeated coumarin derivatives into an “open” inverse opal photonic crystal fluorescence sensing membrane, generating fluorescent products through the specific reaction between vinyl ester-containing coumarin derivatives and cysteine. Kuo and colleagues [17] prepared titanium (TiO_2_) inverse opal films using a self-assembled colloidal crystal template technique to detect ethanol solutions of varying concentrations. Yu et al. [18] incorporated a metal–organic framework (MOF) CDs@UiO-66(OH)_2_ into the matrix of photonic crystal hydrogels for the preparation of MOF/PC hydrogels that exhibit colorful structural colors and emit blue fluorescence. However, the fabrication of these photonic crystals or fluorescent photonic crystals via dip coating or vertical deposition typically demands a longer processing time and is usually carried out on a glass substrate. The rapid construction of fluorescent photonic crystals on various substrates for packaging applications remains a challenge.

The spray-coating approach has the advantages of simplicity, efficiency, and scalability in rapidly constructing optical labels. Wang et al. [19] employed a two-step spray-coating procedure to fabricate waterproof PC coating layers with bright, uniform, and angle-dependent structural colors, enabling the preparation of patterns or full-coverage PC films with excellent chemical and mechanical stability on different substrates. On this basis, the introduction of multicolor fluorescent anti-counterfeiting materials is an efficient way to enhance anti-counterfeiting performance. Carbon quantum dots (CDs), by taking advantage of a wide range of raw material sources, facile preparation methods, and tunable fluorescent emission colors, have exhibited extensive application prospects in the anti-counterfeiting domain [20,21,22]. Hence, multicolor carbon dots-based fluorescent photonic crystal labels represent an effective method of anti-counterfeiting and information storage.

This study puts forward a method for rapid preparation of multicolor dual-mode anti-counterfeiting labels through sequentially spray coating PVA@CDs aqueous suspension and SiO_2_ aqueous suspension. The methods for producing structural color and fluorescence color, the process conditions for constructing photonic crystals by spray coating, and the technical route for generating dual-mode labels were systematically studied. Under optimized circumstances, vivid labels with fluorescence/structural color dual modes were rapidly fabricated. Moreover, the application of these labels in dual-channel information storage and anti-counterfeiting was explored.

## 2. Experimental Section

### 2.1. Materials

Citric acid (Analytical Reagent (AR), ≥ 99.5%), *p*-phenylenediamine (AR, 97%), and polyvinyl alcohol (PVA, Mw ~ 205,000, 97%) were purchased from Aladdin Reagent Co., Ltd. (Shanghai, China). Tetraethyl orthosilicate (TEOS, 98%) was provided by Alpha Aesar (Ward Hill, MA, USA). Ethylenediamine (AR) and ammonia solution (AR, 28–30%) were obtained from Innochem (Beijing, China). Concentrated hydrochloric acid (36%), ethylene glycol (AR), and ethanol absolute (AR) were purchased from Changsha Fenlukou Plastic Chemical Factory (Changsha, China). Deionized water was self-prepared in the laboratory. All the chemicals were used as received without further purification.

### 2.2. Preparation of Multicolor Fluorescent CDs

Multicolor fluorescent CDs were prepared using hydrothermal or solvothermal methods [23]. Specifically, 0.0425 g of *p*-phenylenediamine was dissolved into 40 mL of ethylene glycol with the assistance of a stirrer. A total of 100 μL of hydrochloric acid was added into the above homogeneous solution, and the solution was transferred to a 50 mL polytetrafluoroethylene-lined reaction vessel and reacted at 180 °C for 8 h. After the reaction, a dark brown suspension was obtained and cooled to room temperature naturally. After centrifugation at 11,000 rpm for 10 min to remove the large insoluble particles, the suspension was dialyzed for 24 h. Yellow fluorescent carbon dots (YCDs) suspension was obtained. Through the same operation, green fluorescent carbon dots (GCDs) were prepared by using *p*-phenylenediamine and ethylenediamine as raw materials and water as the solvent. Blue fluorescent carbon dots (BCDs) were synthesized using a conventional hydrothermal method, in which citric acid (1.656 g) served as the carbon precursor, ethylenediamine (530 μL) acted as the nitrogen dopant, and water (40 mL) was employed as solvent.

### 2.3. Preparation of SiO_2_ Microspheres

Firstly, a starting solution was prepared by incorporating 41 mL of ethanol, 7.14 mL of ammonia solution, and 3 mL of deionized water into a 250 mL round-bottom flask. The flask, equipped with a magnetic stirrer, was placed in a water bath for 10 min at 60 °C with a stirring speed of 800 rpm. Successively, 6 mL of TEOS was swiftly added to the round-bottom flask, and the reaction proceeded for 2 h. The solution transformed into a turbid white suspension, signifying the formation of silica colloid nanoparticles. Following the reaction, the product was isolated via centrifugation. Initially, the solution was transferred to a 50 mL centrifuge tube and centrifuged at 6000 rpm for 10 min to eliminate the supernatant. The resultant solid was washed three times with ethanol via ultrasonication and then subjected to evaporative drying in a 60 °C constant-temperature drying oven to obtain uniform silica microsphere solid powder. While keeping the other reaction conditions unchanged, the quantity of ethanol in the starting solution was adjusted to 52 mL and 63 mL to regulate the microsphere dimensions [24].

### 2.4. Preparation of Dual-Mode Anti-Counterfeiting Labels

The diverse carbon quantum dot solution was completely blended with the polyvinyl alcohol solution at a 1:1 ratio, giving rise to a uniform PVA@CDs solution with varying fluorescent colors. Silica microspheres were disseminated in water and diffused into a suspension via ultrasound. The impact of solid content on the assembly of microspheres was explored by preparing silica aqueous solutions with diverse mass fractions (5 wt%, 10 wt%, 15 wt%, 20 wt%, 30 wt%).

### 2.5. Characterization

The morphology of CDs was examined using a transmission electron microscope (TEM, JEM-F200, JEOL, Tokyo, Japan). Fourier transform infrared spectroscopy (FT-IR, TENSOR ΙΙ, Bruker, Billerica, MA, U.S.) and ultraviolet–visible (UV-vis) spectrophotometry (TU-1801, Beijing PUXI General Instrument Co., LTD., Beijing, China) were employed for the characterization of CDs. Photoluminescence (PL) spectra were acquired using fluorescence spectrophotometers (F-4500, Hitachi, Kyoto, Japan). Scanning electron microscopy (SEM, Sigma 300, ZEISS, Oberkochen, Germany) was utilized to analyze the microscopic morphology of SiO_2_ particles and photonic crystals. The reflection spectra of photonic crystals were measured using UV/visible/infrared diffuse reflection testing (Shimadzu, UV-3600, Kyoto, Japan).

## 3. Results and Discussion

**Figure 1:**
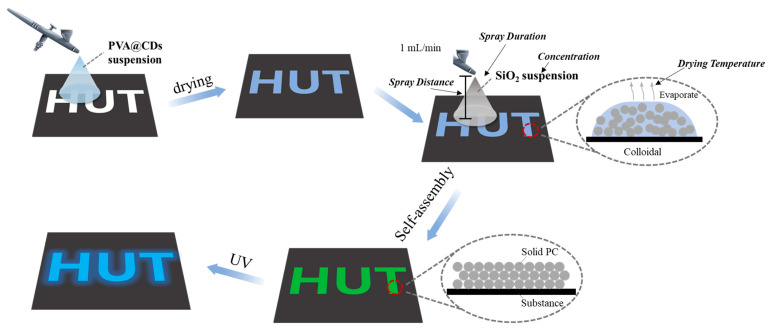
Schematic preparation of PVA@CDs/SiO_2_ fluorescent/structural color dual-mode anti-counterfeiting label.

The preparation process of the fluorescent structural color dual-mode anti-counterfeiting label is depicted in Figure 1. At the outset, the PVA@CDs aqueous suspension was splashed onto the black non-fluorescent paper and then left to dry, forming the patterned PVA@CDs film. Subsequently, a layer of SiO_2_ aqueous solution was sprayed onto the PVA@CDs film. After the evaporation of water, the SiO_2_ microspheres auto-assembled into photonic crystals, and the structural color emerged. The fluorescence pattern constructed by the PVA@CDs was presented when exposed to ultraviolet light.

### 3.1. Characterization of CDs

**Figure 2:**
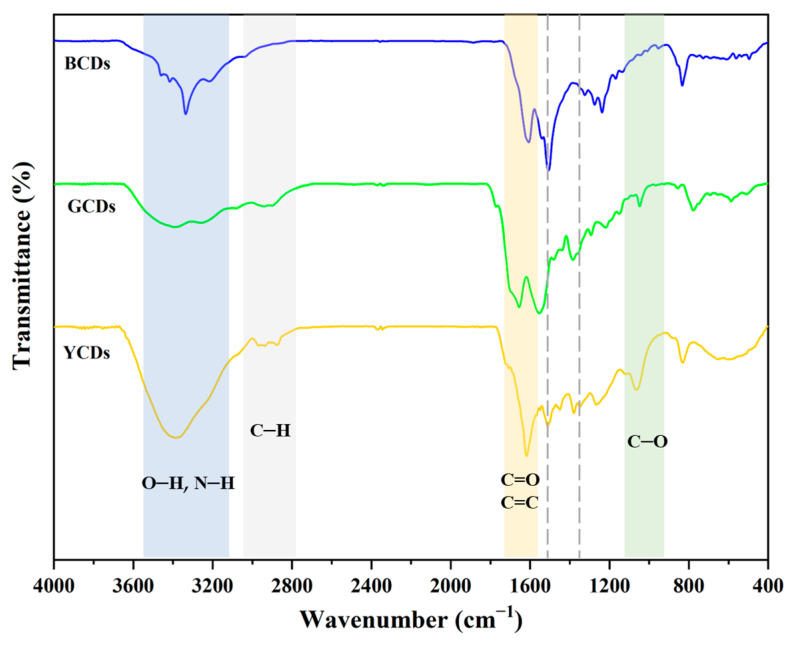
FT-IR spectra of YCDs, GCDs, and BCDs.

FT-IR was utilized to characterize the functional groups on the CD surfaces. As depicted in Figure 2, the FT-IR spectra of the three CDs are similar. The characteristic absorption peaks in the range of 3000–3500 cm^−1^ corresponded to the stretching vibrations of N–H and O–H bonds, indicating the presence of a large number of hydroxyl groups on the surface, which contributes to the water solubility and biocompatibility of the CDs. The stretching vibrations of C–H bonds resulted in characteristic absorption peaks around 2750–3000 cm^−1^ for the CDs, indicating the presence of aliphatic hydrocarbon bonds, which may originate from the precursor or alkyl chains introduced during the synthesis process. Additionally, the absorption peak in the range of 1700–1750 cm^−1^ due to the stretching vibrations of C=O bonds indicates the presence of carboxyl or carbonyl groups, likely resulting from the oxidation process of the carbon precursor. The significant absorption peak around 1600 cm^−1^, caused by the stretching vibrations of C=C bonds, suggests the presence of aromatic ring structures, which enhance the fluorescence properties and stability of the CQDs. Furthermore, the absorption peak in the range of 1500–1600 cm^−1^ due to the bending vibrations of N–H bonds indicates the introduction of amino groups for surface modification [25]. The absorption peak near 1050 cm^−1^ corresponds to the stretching vibrations of C–O bonds, indicating the presence of alcohol, ether, or ester structures. These functional groups also contribute to the water solubility and biocompatibility of the CQDs. These results indicate that the three types of carbon quantum dots are composed primarily of C, H, O, and N elements, and contain hydroxyl and amino functional groups, making them water-soluble. In the 1380 to 1520 cm^−1^ region, the GCDs and YCDs exhibit a prominent peak corresponding to the vibration of the benzene ring skeleton, whereas the BCDs do not display this characteristic peak. This discrepancy arises from the fact that GCDs and YCDs are synthesized using *p*-phenylenediamine, which imparts the distinctive benzene ring peak at these carbon sites [26].

**Figure 3:**
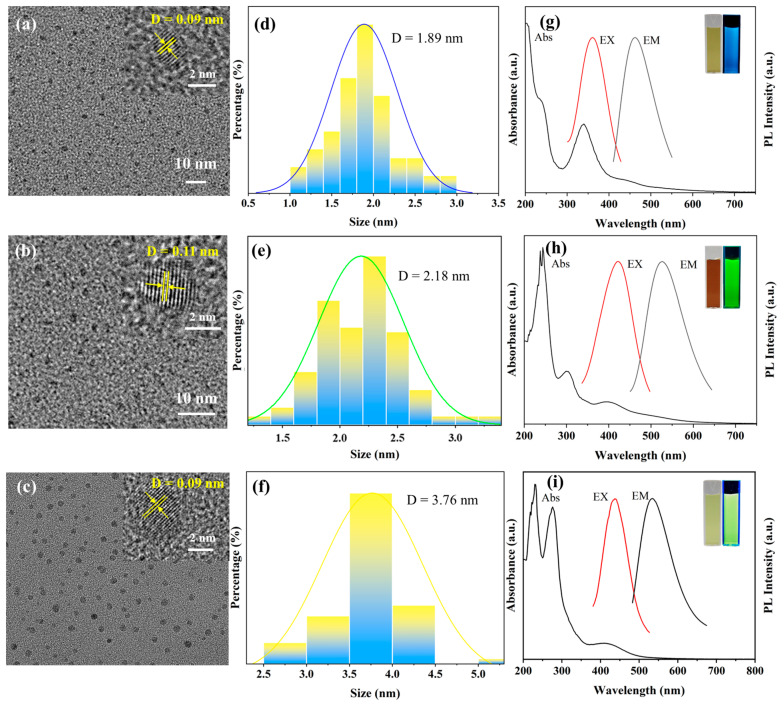
TEM images of BCDs (**a**), GCDs (**b**), and YCDs (**c**). Average sizes of BCDs (**d**), GCDs (**e**), and YCDs (**f**). “D” denotes the mean particle diameter. The UV-Vis absorption spectra and excitation–emission spectra of BCDs (**g**), GCDs (**h**), and YCDs (**i**). Insets: photographs of carbon quantum dots under sunlight (left) and 365 nm UV lamp irradiation (right).

The morphology characterization of the three CDs was conducted using transmission electron microscopy, and the results are shown in Figure 3a–c. The three types of CDs exhibit approximately spherical nanoparticles with good dispersion and no aggregation observed. The average particle sizes of the YCDs, GCDs, and BCDs were measured to be 3.76 nm, 2.18 nm, and 1.89 nm, respectively. TEM images showed that all CDs had lattice fringes with good resolution, and their lattice spacing was 0.09 nm, 0.11 nm, and 0.09 nm, respectively, which could be attributed to the (100) diffraction facet of graphite. The fluorescent color of the CDs shifts from blue to yellow, possibly due to the increase in particle size and the degree of graphitization [27,28].

The optical properties of the CDs were examined using a UV-vis spectrophotometer and a fluorescence spectrometer. Figure 3g–i depict the UV-vis absorption spectra of the three CDs, revealing strong absorption peaks in the 275–300 nm range, which are attributed to π→π* transitions in the aromatic sp^2^ domains, mainly involving functional groups such as C=C and C=N. Moreover, the YCDs exhibit a shoulder peak at 428 nm, and GCDs display a shoulder peak at around 426 nm, which may be due to defect-state n→π* transitions, mainly involving functional groups such as C–O and C=O. This indicates differences in chemical composition and structure among the different CDs, which may be due to the variations in their synthesis methods or raw materials. As shown in Figure 3g–i, the maximum excitation wavelength of the YCDs, GCDs, and BCDs is 482 nm, 421 nm, and 360 nm, respectively. Correspondingly, the maximum fluorescence emission wavelength is 536 nm, 527 nm, and 462 nm, respectively. The illustrations of Figure 3g–i present the images of the prepared CDs solutions under visible light and 365 nm UV light irradiation. Under normal lighting conditions, the prepared carbon quantum dot solutions appear translucent yellowish or yellowish-brown. However, under the illumination of a 365 nm UV lamp, the YCDs, GCDs, and BCDs exhibit yellow, green, and blue fluorescence, respectively.

**Figure 4:**
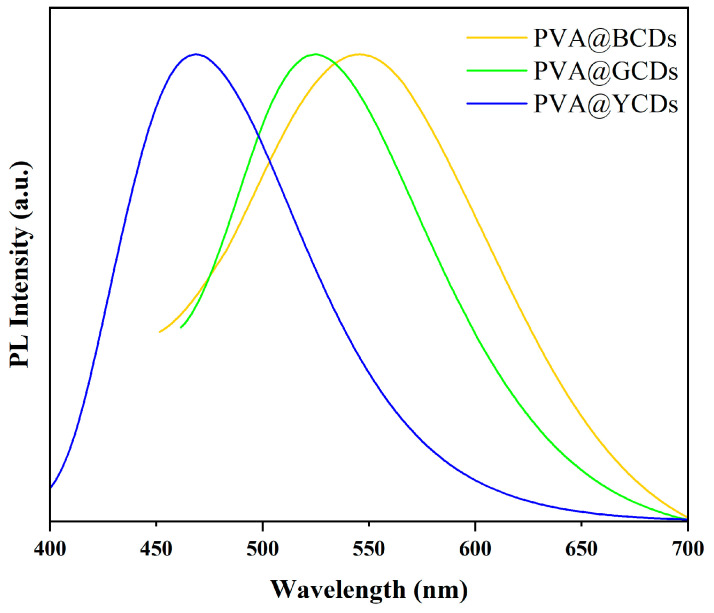
Fluorescence spectra of PVA@YCDs, PVA@GCDs, PVA@BCDs.

The coating effect of PVA helps maintain the monodispersity of CDs, enhances their stability in solution, slows down fluorescence quenching, and thereby improves the fluorescence performance of CDs. This is suitable for applications requiring long-term stable fluorescence. To maintain the luminescence of CDs in solid-state labels, PVA was chosen as the substrate for preparing hybrid materials to ensure the monodispersity and prevent the aggregation quenching of CDs. The fluorescent spectra of PVA@CDs are recorded in Figure 4. The figure displays three fluorescence emission peaks at 468 nm, 525 nm, and 546 nm. The fluorescent emission peaks of PVA@BCDs, PVA@GCDs, and PVA@YCDs have no shift in comparison with those of the BCDs, GCDs, and YCDs solutions. It was demonstrated that the PVA substrate has no influence on the luminescence of CDs.

### 3.2. Characterization of Photonic Crystals

**Figure 5:**
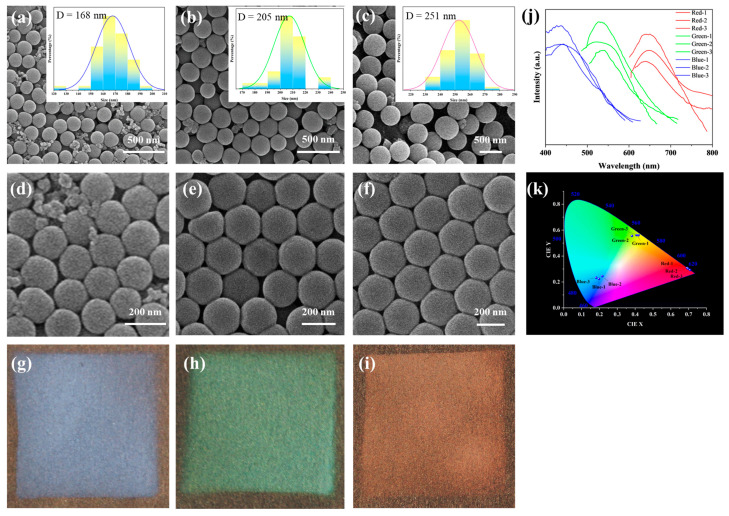
SEM images and particle size distribution of microspheres with average diameters of 168 nm (**a**), 228 nm (**b**), and 263 nm (**c**). SEM images of photonic crystals with blue (**d**), green (**e**), and red (**f**) structure color. Optical images of photonic crystals with blue (**g**), green (**h**), and red (**i**) structure color. Reflection spectra (**j**) and CIE chromaticity diagrams (**k**) of blue, green, and red photonic crystals.

The microstructures of assembled SiO_2_ particles were examined by SEM, as shown in Figure 5a–c. All three types of silica particles exhibit well-defined spherical morphology, with average particle sizes of 263 nm, 228 nm, and 168 nm, respectively. By varying the volume of ethanol (41 mL, 52 mL, and 63 mL) while keeping other reaction conditions constant, the particle size of silica microspheres is controlled [29]. The results show that the final size had a negative correlation with the volume of ethanol used in the formulation.

SiO_2_ microspheres with the above three particle sizes were prepared into 15% suspension. After the application of the spray, the SiO_2_ microspheres with uniform particle size are densely packed to form photonic crystals, as presented in Figure 5d–f. The photonic crystals are piled up in a hexagonal closed-packed arrangement, which leads to the display of structural color due to the photon bandgap effect. In Figure 5g–i, the structural colors corresponding to red, green, and blue are shown. The optical properties of the prepared photonic crystals were characterized by the reflection spectrum. As shown in Figure 5j, the reflection peaks of the photonic crystals prepared by SiO_2_-168, SiO_2_-228, and SiO_2_-263 microspheres were located at 437 nm, 541 nm, and 659 nm, respectively. Furthermore, variations in intensity can be discerned in the reflectance spectrograms at different locations of the sample, possibly attributed to the irregular surface of the paper and uneven hand spraying. At the same time, Figure 5k is the Commission Internationale de I’Eclarage (CIE) chromaticity diagram drawn by the reflection spectrum, which can be seen to be consistent with the structural color seen by the naked eye. The optical properties of photonic crystals are mainly related to their photonic bandgaps, the center wavelength of which can be computed by using the Bragg equation (Equation (1)).

(1) $$\lambda =2n_{eff}d_{hkl}$$

In Equation (1), λ represents the center wavelength of the photonic bandgap, and n_eff_ indicates the effective refractive index of the lattice, which can be calculated using Equation (2). d_hkl_ represents the lattice spacing of the crystal in the (hkl) direction, which can be calculated based on the diameter of the silica microspheres. For face-centered cubic-structured silica microspheres, the calculation formula is provided in Equation (2).

(2) $$n_{eff}={\left[n_{{SiO}_{2}}^{2}f+n_{air}^{2}\left(1-f\right)\right]}^{\frac{1}{2}}$$

In Equation (2), $n_{{SiO}_{2}}$ and $n_{air}$ represent the refractive indices of silica and air, respectively, where $n_{{SiO}_{2}}$= 1.45 and $n_{air}$ = 1. f denotes the filling fraction of silica, which is derived as f = 0.74 based on the face-centered cubic structure of silica. According to the calculation of the Bragg equation, the center wavelengths of the photonic bandgaps for the three fabricated photonic crystals are determined as 453 nm, 553 nm, and 677 nm. The corresponding emitted colors at these wavelengths are blue, green, and red, which are in line with the structural colors observed in Figure 5d–f. The calculated values are close to the spectral test results. The shift of the reflectance peaks may be attributed to the non-uniform size and non-uniform accumulation of SiO_2_ microspheres.

**Figure 6:**
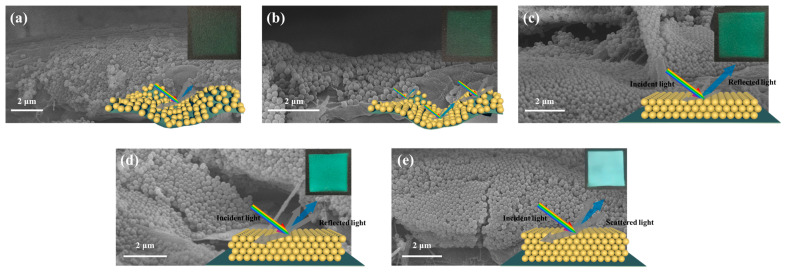
SEM images, physical diagram (top right), and schematic representations (bottom right) of spray coating effects of dispersion liquids with concentrations of 5% (**a**), 10% (**b**), 15% (**c**), 20% (**d**), and 30% (**e**).

The weight fraction of SiO_2_ in the spray solution plays a crucial role in determining the microsphere spacing and is also a key factor in influencing the quality of the structure color. Different mass fractions (5%, 10%, 15%, 20%, and 30%) of the SiO_2_ microsphere (with a size of 168 nm) solutions are used for spraying. The spray coating effects on black paper are presented in Figure 6. The microspheres assembled by spray coating a dispersion liquid with a concentration of 5% and 10% SiO_2_ show structural coloration with a lighter hue, as shown in Figure 6a,b. The low concentration led to the particles initially penetrating the gaps between the fibers, which is not sufficient to form a continuous membrane, thereby resulting in a weak reflection intensity. In contrast, for the dispersion liquid with a concentration of 15% SiO_2_, the concentration is considered appropriate, giving clear and deep colors; as shown in Figure 6c, the SiO_2_ particles are arranged neatly. It can be noticed that the photonic crystals assembled by spray coating a dispersion liquid with a higher concentration of 20% silica show unsatisfactory color saturation, appearing whitish, as shown in Figure 6d. The thickness of the SiO_2_ accumulation increases. When the concentration of the microspheres is further increased to 30%, the structural color almost disappears, showing only the white color of the silica; as shown in Figure 6e, the SiO_2_ particles are arranged periodically, and the accumulation thickness further increases. This phenomenon is due to the excessive concentration within the photonic crystals, resulting in increased light scattering within the crystals. The enhanced light scattering interferes with the formation of structural colors, resulting in a dimmer appearance and even whitening. Moreover, higher concentrations may induce disorderliness in the photonic crystal structure, disrupting the regular arrangement of particles and consequently affecting the photonic crystal’s coloration [30]. Therefore, it can be deduced that the optimal spray coating concentration is 15%.

In addition to the concentration of microspheres, spray distance, spray velocity, and spray duration are also critical factors that affect the quality of the structure color. These parameters influence the spacing and packing thickness of the microspheres, which in turn determine the arrangement order and light scattering efficiency. The spacing of the microspheres needs to be optimal to achieve a tightly ordered photonic crystal structure. Additionally, if the packing thickness is insufficient, it is unable to create a continuous structural color, while an excessive packing thickness leads to serious light scattering which turns the film whitish. The self-assembly process of microspheres is influenced by the strength of the Brownian motion and solvent volatilization, both of which are temperature-dependent. Optimal assembly quality is attained within a specific temperature range. Hence, the spray parameters of the SiO_2_ photonic crystals were optimized by orthogonal experiment, as shown in Table 1. During the experiment, a small spraying distance facilitates the formation of flowing droplets on the substrate. Conversely, when the distance is too large, excessive material may be sprayed outside the base material, hindering accumulation layer formation. If the spraying time is insufficient, the particle accumulation thickness will be inadequate; conversely, an excessively long spraying time will result in excessive accumulation thickness. High drying temperatures can cause microspheres to become fixed on the substrate without proper assembly, while low temperatures may lead to insufficient assembly power. The appropriate spray conditions were controlled to a spray velocity of 1 mL/min, a spray distance of 10 cm, a spray duration of 3 s, a particle concentration of 15%, and an assembly temperature of 20 °C.

### 3.3. Multicolor PVA@CDs/SiO_2_ for Dual-Mode Anti-Counterfeiting Labels

**Figure 7:**
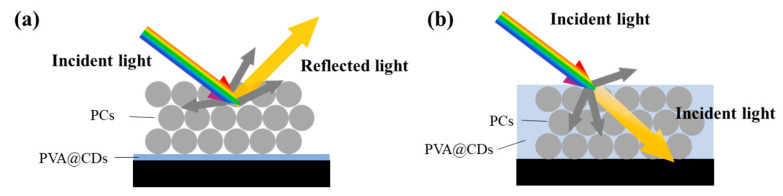
Label diagram of PVA@CDs aqueous suspension sprayed first (**a**) and SiO_2_ aqueous suspension sprayed first (**b**).

The PVA@CDs/SiO_2_ composite was prepared using the spraying method, and the sequence of spraying the two solutions influenced the assembly of the composite. Figure 7 depicts the different assembly processes resulting from the two spraying methods. Figure 7a shows the procedure of spraying and drying the PVA@CDs solution to form a PVA film, which served as a substrate for SiO_2_ microsphere assembly. Upon exposure to water, some microspheres may get embedded in the PVA film, but this does not entirely hinder the self-assembly of the microspheres into photonic crystals. In contrast, when the SiO_2_ aqueous suspension is sprayed first, the microspheres self-assemble into an opal structure with numerous gaps. Subsequently spraying the PVA@CDs solution allows it to penetrate these gaps (Figure 7b). The refractive index of SiO_2_ is about 1.50, and the refractive index of PVA is between 1.49 and 1.52. Since the refractive index of SiO_2_ is similar to that of PVA, light in it will only pass through and is difficult to reflect, and the structural color will disappear. Therefore, we first sprayed PVA@CDs aqueous solution and then sprayed SiO_2_ aqueous solution on PVA@CDs thin film to ensure the structural color of the photonic crystal.

**Figure 8:**
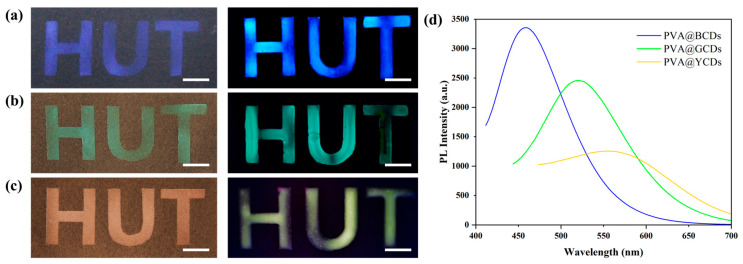
Photograph of dual-mode anti-counterfeiting label patterns synthesized with PVA@BCDs/SiO_2_-168 (**a**), PVA@GCDs/SiO_2_-228 (**b**), and PVA@YCDs/SiO_2_-263 (**c**) under natural light (scale bar of 1 cm) and UV lamp (right column, scale bar of 1 cm). (**d**) Fluorescence intensity test of dual-mode anti-counterfeiting label patterns.

Figure 8 shows a photograph of the anti-counterfeiting labels synthesized with SiO_2_ particles of average diameters ranging from 168 nm to 228 nm and 263 nm along with PVA@CDs under natural light and a UV lamp. The components of the blue/blue (Figure 8a), green/green (Figure 8b), and red/yellow (Figure 8c) dual-mode labels are PVA@BCDs/SiO_2_-168, PVA@GCDs/SiO_2_-228, and PVA@YCDs/SiO_2_-263, respectively. The fluorescence spectra of the three labels are depicted in Figure 8d. Due to variations in the quantum yield of the carbon dots, the fluorescence intensity of the labels varies, corresponding to their visual brightness. These two-mode labels display three different saturated structure color “HUT” patterns alongside three different vibrant fluorescent color “HUT” patterns. The preparation method does not impose constraints on the particle size and CD type of the microsphere, providing ample flexibility for adjusting the color of the dual-mode label.

**Figure 9:**
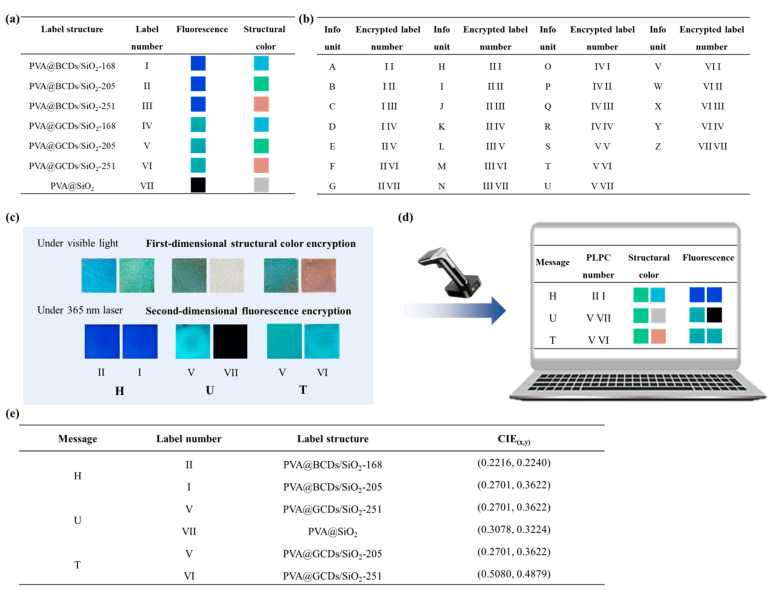
Encoding units of different PVA@CDs/SiO_2_ labels (**a**). Coding schedule for 26 letters (**b**). Dual-mode encoding information for encryption of “HUT” (**c**). The matching of label numbers based on structural color information and fluorescence color information for decoding information (**d**). CIE chromaticity coordinates corresponding to “HUT” (**e**).

Fluorescent/structure color dual-mode labels can be used for information storage with enhanced security. As proof of theory, three structure colors (blue, green, and red) and two fluorescent colors (blue and green) were chosen to construct an encoding system. As shown in Figure 9a, seven different labels composed of PVA@BCDs/SiO_2_-168, PVA@BCDs/SiO_2_-228, PVA@BCDs/SiO_2_-263, PVA@GCDs/SiO_2_-168, PVA@GCDs/SiO_2_-228, PVA@GCDs/SiO_2_-263, and PVA@SiO_2_ (without structure color and fluorescence) act as the encryption unit and were numbered “Ι”, “ΙΙ”, “ΙΙΙ”, “ΙV”, “V”, “VΙ”, and “VΙΙ”. The defined coding schedule for 26 letters is depicted in Figure 9b. Specific combinations of two labels determine a character. For instance, “A” is represented by “Ι” and “Ι”.

The complete encoding and decoding process consists of four steps. The English acronym “HUT” for Hunan University of Technology was selected as a sample to explain the procedure. Firstly, following the encryption rules (Figure 9b), the sequence of label numbers (II, I, V, VII, V, VI) was identified. Secondly, based on the structural characteristics of the labels (Figure 9a), six labels were prepared by sequentially spraying different PVA@CDs solutions and SiO_2_ aqueous solutions. Through these two steps, the message was transformed into a label array. To decode the information, it is essential to obtain the structural color information of the labels as the first-dimensional decoding data, and the fluorescent color of the labels as the second-dimensional decoding data. The numbers of the labels were determined by integrating two-dimensional information. Finally, the decoding information “HUT” was obtained in accordance with the encryption rules.

**Figure 10:**
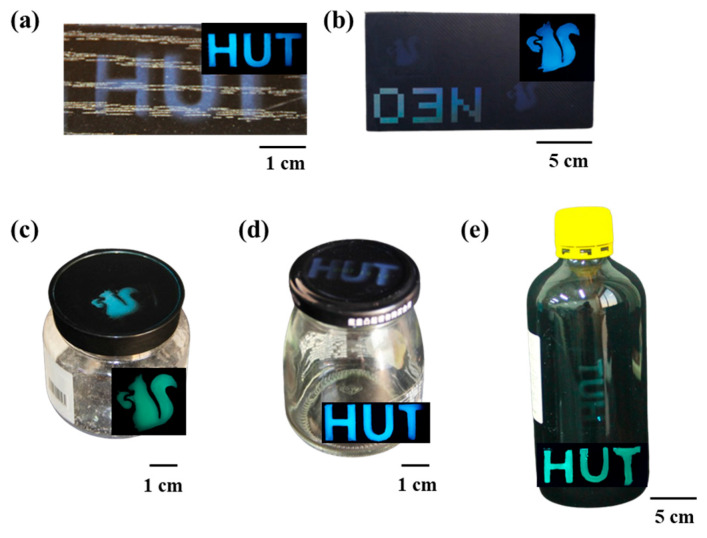
Coloration effects of dual-mode anti-counterfeiting labels sprayed on different substrates: wood (**a**), paper (**b**), plastic (**c**), metal (**d**), and glass (**e**).

To evaluate the suitability of the dual-mode label construction, five common packaging materials were selected as the objects for research. In Figure 10, images of the dual-mode label anti-counterfeiting patterns on the surfaces of wood, paper, plastic, metal, and glass. The photos taken in natural light show saturated and patterned blue structural color HUT characters and squirrel patterns. Under UV light, these patterns display bright fluorescent colors. The anti-counterfeiting method shows excellent adaptability to various types of packaging. The structural color based on microsphere assembly is less reproducible than conventional printed patterns and also shows the hidden fluorescence characteristics of carbon dots. The dual-mode label can effectively act as an anti-counterfeiting measure due to the complexity of replication without key information.

## 4. Conclusions

The proposed method involves the rapid construction of fluorescence/structural color dual-mode optical labels by spraying PVA@CDs aqueous suspension along with SiO_2_ aqueous suspension. When the spraying distance is 10 cm, the spraying time lasts for 3 s, and the drying temperature is controlled at 20 °C. A SiO_2_ aqueous suspension with a mass fraction of 15% can be sprayed to obtain a photonic crystal with a good assembly effect. The adjustable structural color and luminescence color of CDs allow the dual-mode tags to have colorful characteristics, providing large information storage capacity and security. Compared to single-mode optical anti-counterfeiting technologies, the dual-mode tags’ combination of structural color and fluorescence is more difficult to copy, which provides a higher level of security. In the developed sequential spraying method, the initial PVA@CDs layer not only confers a stable invisible fluorescent characteristic to the label but also provides a suitable interface for subsequent silica assembly. As a consequence, the dual-mode anti-counterfeiting label demonstrates excellent adaptability for various substrates. The spray self-assembled microspheres also reduce the time and smooth the interface requirements of vertical deposition and dip coating. However, current label production is restricted in information storage capability and requires additional protective layers or processing technologies to guarantee durability in practical applications.

## Figures and Tables

**Table 1:** Orthogonal experimental table for optimization of SiO_2_ photonic crystal spraying parameters.

Test Number	Concentration	Spray Distance	Spray Duration	Drying Temperature
1	5%	3 cm	1 s	10 °C
2	10%	5 cm	3 s	20 °C
3	15%	7 cm	5 s	30 °C
4	20%	10 cm	7 s	40 °C
5	30%	15 cm	10 s	50 °C

## Data Availability

The original contributions presented in the study are included in the article; further inquiries can be directed to the corresponding author.
